# Carbamazepine for prevention of chemotherapy-induced nausea and vomiting: a pilot study

**DOI:** 10.1590/1516-3180.2014.1323600

**Published:** 2014-04-14

**Authors:** Thaiana Aragão Santana, Felipe Melo Cruz, Damila Cristina Trufelli, João Glasberg, Auro Del Giglio

**Affiliations:** I MD. Oncology Fellow, Faculdade de Medicina do ABC (FMABC), São Paulo, Brazil; II MD, MSc. Attending Physician, Discipline of Oncology, Faculdade de Medicina do ABC (FMABC), São Paulo, Brazil; III MD. Attending Physician, Discipline of Oncology, Instituto do Câncer do Estado de São Paulo (ICESP), Faculdade de Medicina da Universidade de São Paulo (FMUSP), São Paulo, Brazil; IV MD, MSc, PhD. Titular Professor, Discipline of Oncology, Faculdade de Medicina do ABC (FMABC), São Paulo, Brazil

**Keywords:** Antiemetics, Vomiting, Nausea, Carbamazepine, Prevention and control [subheading], Antieméticos, Vômito, Náusea, Carbamazepina, prevenção & controle

## Abstract

**CONTEXT AND OBJECTIVE:**

Nausea and vomiting are major inconveniences for patients undergoing chemotherapy. Despite standard preventive treatment, chemotherapy-induced nausea and vomiting (CINV) still occurs in approximately 50% of these patients. In an attempt to optimize this treatment, we evaluated the possible effects of carbamazepine for prevention of CINV.

**DESIGN AND LOCATION:**

Prospective nonrandomized open-label phase II study carried out at a Brazilian public oncology service. METHODS: Patients allocated for their first cycle of highly emetogenic chemotherapy were continuously recruited. In addition to standard antiemetic protocol that was made available, they received carbamazepine orally, with staggered doses, from the third day before until the fifth day after chemotherapy. Considering the sparseness of evidence about the efficacy of anticonvulsants for CINV prevention, we used Simon's two-stage design, in which 43 patients should be included unless overall complete prevention was not achieved in 9 out of the first 15 entries. The Functional Living Index-Emesis questionnaire was used to measure the impact on quality of life.

**RESULTS:**

None of the ten patients (0%) presented overall complete prevention. In three cases, carbamazepine therapy was withdrawn because of somnolence and vomiting before chemotherapy. Seven were able to take the medication for the entire period and none were responsive, so the study was closed. There was no impact on the patients' quality of life.

**CONCLUSION:**

Carbamazepine was not effective for prevention of CINV and also had a deleterious side-effect profile in this population.

## Abstract (pt)

**CONTEXTO E OBJETIVO:**

Náusea e vômito são inconvenientes importantes para pacientes submetidos a quimioterapia. A despeito do tratamento preventivo padrão, náuseas e vômitos induzidos por quimioterapia (NVIQ) ocorrem em aproximadamente 50% dos pacientes. Na tentativa de otimizar este tratamento, avaliamos os possíveis efeitos da carbamazepina na prevenção de náuseas e vômitos induzidos por quimioterapia.

**TIPO DE ESTUDO E LOCAL:**

Estudo fase II, prospectivo, não randomizado, aberto, realizado em um serviço público brasileiro de oncologia.

**MÉTODOS:**

Recrutaram-se continuamente pacientes alocados para o primeiro ciclo de quimioterapia altamente emetogênica. Além do protocolo anti-emético padrão disponibilizado, os pacientes receberam carbamazepina, por via oral, em doses escalonadas, a partir do terceiro dia anterior até o quinto dia após a quimioterapia. Dada a escassa evidência de eficácia dos anticonvulsivantes na prevenção de NVIQ, adotamos o desenho de Simon em duas fases, que deveria incluir 43 pacientes a não ser que prevenção completa global não fosse alcançada em 9 dos primeiros 15 participantes. O questionário "Functional Living Index-Emesis" foi usado para avaliar o impacto na qualidade da vida.

**RESULTADOS:**

Nenhum dos 10 pacientes (0%) apresentou prevenção completa global. Três tiveram a carbamazepina suspensa por sonolência e vômito antes da quimioterapia. Sete foram capazes de tomar a medicação por todo o período proposto e nenhum obteve resposta, sendo então interrompido o estudo. Não houve impacto na qualidade da vida.

**CONCLUSÃO:**

Carbamazepina não foi efetiva para prevenção de NVIQ e apresentou perfil deletério de efeitos adversos nesta população.

## INTRODUCTION

Nausea and vomiting are major inconveniences for patients undergoing cancer therapy. These symptoms, which are both common and stressful, are reported by almost half of such patients, either as a consequence of the illness itself, or as a treatment side effect.^1^ They have a strikingly negative impact on these patients' functional, emotional, social and nutritional status, ultimately leading to impairment of quality of life.^2^ ^,^ ^3^

Several factors have been implicated in development of chemotherapy-induced nausea and vomiting (CINV), i.e. the intrinsic emetogenicity of some chemotherapeutic agents, either alone or in combination.^2^ ^,^ ^4^ ^,^ ^5^ CINV is classified according to the timing of the occurrence: acute - occurs and resolves within the first 24 hours after chemotherapy; or delayed - occurs after the first 24 hours after chemotherapy administration.^5^ ^,^ ^6^ Because of the severity of the acute phase, this has been more often targeted in therapeutic intervention studies. However, although the symptoms in the delayed period are less marked than those observed in acutely started nausea and vomiting, its course can be more protracted, resulting in poor hydration and nutrition control, in addition to poor performance status.^7^ ^,^ ^8^

According to the American Society of Clinical Oncology (ASCO) guidelines for antiemetic management in cancer patients, the standard preventive treatment for CINV is based on selective 5-HT3-receptor antagonists (5HT3AR), corticosteroids and neurokinin-1-receptor antagonists (NK1AR), combined to match the intensity of chemotherapy-induced vomiting.^9^ ^-^ ^14^ Despite the use of such therapy, approximately 50% of patients still present CINV.^15^

However, treatment with these three drugs has a high cost and some of them are unavailable in centers where healthcare funding is provided by the government. In most Brazilian cities, for instance, cancer patients covered by the national healthcare system have no sponsorship for use of either 5HT3RA or NK1RA while being treated in outpatient clinics. In this setting, because medicines are unaffordable for most of these patients, CINV control is even more poorly accomplished.

Efforts to improve CINV prevention have led to investigation of other drugs that can be added to the standard treatment, thus retaining the effectiveness of antiemetics at lower cost. With this purpose, Cruz et al. developed a phase II randomized trial in which they demonstrated that the preventive antiemetic action of gabapentin increased when it was included in the combined treatment during the first cycle of highly emetogenic chemotherapy. Although the mechanism of action of gabapentin has not been well clarified, this trial suggested that anticonvulsant drugs could be promising for prevention of CINV.^16^ Carbamazepine was reported by Strohscheer and Borasio as being completely successful in combating CINV in one patient with meningeal carcinomatosis.^17^

## OBJECTIVE

In the present study, the primary objective was to evaluate the possible effects of carbamazepine in preventing nausea and vomiting induced by highly emetogenic chemotherapy. The secondary objective was to evaluate the side effects of this treatment and its possible influence on patients' quality of life.

## METHODS

Patients were continuously recruited at a Brazilian public oncology service. Between December 2011 and March 2012, patients ≥ 18 years of age, with Eastern Oncology Group (ECOG) grade ≤ 2, who were scheduled to receive their first cycle of moderately or highly emetogenic chemotherapy (defined as cisplatin, doxorubicin or epirubicin at doses higher than 60 mg/m^2^, 50 mg/m^2^ and 50 mg/m^2^ respectively) were selected to enter the study group after signing an informed consent form. The exclusion criteria were as follows: presence of concurrent diseases as possible causes of nausea and vomiting; concomitant radiotherapy; regular use of opioids, corticosteroids, benzodiazepines, tricyclic antidepressants or cannabinoids before chemotherapy; and failure to attend follow-up visits. This study was approved by our local ethics committee.

All patients received the standard antiemetic treatment that was available, which was based on intravenous ondansetron (8 mg), dexamethasone (10 mg) and ranitidine (50 mg) before chemotherapy infusion, followed by oral dexamethasone (4 mg), twice a day on days 2 and 3. Carbamazepine was added to this treatment in accordance with the following schedule: one tablet (200 mg) four times a day on the third day before chemotherapy; one tablet twice a day on the second day before chemotherapy; and one tablet three times a day starting on the day before chemotherapy and continuing until the fifth day after chemotherapy.

The patients were asked to record vomiting episodes on diary cards, starting on the day of chemotherapy infusion (0 h) and continuing until the morning of day 6 (120 h). In case of need, participants were free to take a "rescue therapy", and the components of this were also to be recorded. The rescue medications included 5HT3RA, phenothiazines, butyrophenones and domperidone.

Complete prevention of nausea and vomiting was defined as the absence of any episode of these events and no use of rescue medication. Symptoms were defined as acute (occurring during the first 24 hours after chemotherapy), delayed (occurring from day 2 to day 5 after chemotherapy) or overall (occurring over the full 120 hours of the study).^18^ ^,^ ^19^ Since NK1AR is not provided to Brazilian patients through the public healthcare system, this drug was not used. 

The patients filled out the Functional Living Index-Emesis (FLIE) questionnaire on days 1 and 6, as backing for measurement of the impact of CINV on their quality of life.^18^ Adverse events were recorded up to the visit on day 6 after chemotherapy. 

Considering that there is a lack of evidence regarding the efficacy of anticonvulsants for CINV prevention, Simon's optimal two-stage design^19^ was used. Based on the previous trial by Cruz et al.^16^ at our institution, which resulted in a 42.5% control rate in the placebo group, we used a control rate < 50% as the null hypothesis to be tested and a rate > 70% as the alternative hypothesis, since the previous trial showed a 20% benefit from associating gabapentin. The initial end point to be pursued was full control for a minimum of 9 patients out of the 15 in the first stage of the study. Fulfillment of this aim would maintain accrual of an additional 28 patients as a second phase. At the end of the study, in accordance with its design, achievement of complete control in 27 or more patients would be required to reject the null hypothesis. This design had 80% power while maintaining an α error rate of 5%. Summary statistics, including means, standard deviations and medians, were generated for continuous variables.^19^ ^,^ ^20^

## RESULTS

Twelve patients were enrolled in the first phase of the study. These patients were continuously recruited and were all women who had been assigned to receive a highly emetogenic chemotherapeutic regimen of doxorubicin and cyclophosphamide for treatment of breast cancer (100%). The mean age was 48 years (Table 1). Two patients were excluded because of refusal to sign the consent statement and opioid use during the study period. Three patients could not complete the intended treatment due to the advent of untoward manifestations before starting chemotherapy: two presented vomiting before chemotherapy infusion and one had excessive (grade 3) somnolence. 

**Table 1.** Patients' characteristics

	All patients (n = 10)
Gender	
Female	10 (100%)
Mean age (years)	48
ECOG score	
0	10 (100%)
1	0 (0%)
2	0 (0%)
Type of chemotherapy	
AC	10 (100%)

ECOG = Eastern Oncology Group

AC = adriamycin (doxorubicin) and cyclophosphamide

The primary objective of the study was not accomplished, since seven patients proved unresponsive to their properly taken trial treatment throughout the intended time span. Since this finding precluded the alternative hypothesis (> 70%), the study was discontinued. Other adverse events are displayed in Table 2. Acute complete control failed in all cases. One patient (10%) achieved acute control of vomiting and this same patient also achieved delayed complete protection (10%). Rescue medications were necessary for nine patients (90%) (Table 3).

**Table 2.** Adverse events

Type of toxicity	n (%)
Anorexia	
Grade 1	1 (10%)
Grade 2	1 (10%)
Grade 3	0
Constipation	
Grade 1	2 (20%)
Grade 2	0
Grade 3	0
Dysgeusia	
Grade 1	0
Grade 2	2 (20%)
Grade 3	0
Dyspepsia	
Grade 1	1 (10%)
Grade 2	0
Grade 3	0
Fatigue	
Grade 1	3 (30%)
Grade 2	1 (10%)
Grade 3	0
Somnolence	
Grade 1	4 (40%)
Grade 2	3 (30%)
Grade 3	1 (10%)
Dizziness	
Grade 1	1 (10%)
Grade 2	3 (30%)
Grade 3	1 (10%)

**Table 3.** Complete control over nausea and vomiting and use of rescue medication, according to study phase

	Acute	Delayed	Overall
Control over nausea and vomiting	0	1 (10%)	0
Use of rescue medication	9 (90%)	5 (50%)	9 (90%)

There was no impact on patients' quality of life, according to results from the FLIE questionnaire. 

## DISCUSSION

The present study showed that carbamazepine was ineffective for CINV prevention. All patients failed to attain complete control of their symptoms, and most (90%) needed rescue medications. Moreover, carbamazepine was associated with more side effects than expected, such as somnolence and dizziness, in addition to induction of vomiting before chemotherapy infusion onset in approximately 20% of the patients.

At our institution, Cruz et al. conducted a prospective, double-blinded, placebo-controlled study that showed that adding gabapentin to the standard preventive antiemetic treatment for moderately and highly emetogenic chemotherapy was effective (65% vs. 42.5%; P = 0.04).^16^ Their trial demonstrated that the acute complete control rate and use of gabapentin were independent factors for achieving an overall complete response. These results suggested that gabapentin might be a cost-effective well-tolerated alternative to NK1RA, with a safety profile similar to that of the placebo. However, despite the anticonvulsant action of carbamazepine, this drug failed to relieve symptoms in our study, given that nausea and vomiting were not curbed in any of the participants, and that this drug was even hazardous to 30% of them because it elicited grade II dizziness and somnolence. The only patient who achieved delayed complete control had previously achieved acute control of vomiting. Although the antiemetic mechanism of action of gabapentin remains unknown, the reason for the different results from these two studies, which were carried out with similar methods in overlapping groups of subjects, could be that over the therapeutic concentration range, carbamazepine does not affect the GABA or glutamate-mediated neuronal pathways.^21^ GABA-related drugs need to be reassessed in future trials in order to establish their value as adjuvants in dealing with CINV.

Strohscheer and Borasio reported on a patient who achieved complete cessation of therapy-refractory nausea and vomiting through use of carbamazepine.^17^ This patient presented meningeal carcinomatosis and paroxysmal syndrome as the causes of the symptoms. Our patients did not have any evidence of central nervous system involvement and failed to control nausea and vomiting when treated with carbamazepine. Further studies will be needed in order to better understand why carbamazepine hinders nausea and vomiting in meningeal carcinomatosis patients but, contradictorily, is disappointing in unaffected patients.

Phase III studies have already shown that benzodiazepines and olanzapine are effective for improving control over CINV when combined with standard treatment based on 5HT3RA and corticosteroids.^22^ ^-^ ^24^

In the present study, we used Simon's optimal two-stage design because of the lack of evidence regarding carbamazepine use for preventing CINV. This is an innovative methodology for investigating nausea and vomiting, and its main advantage is that it avoids exposure of too many patients to an ineffective treatment. It should be reappraised in future pilot trials concerning prevention of CINV.^19^ ^,^ ^25^ Furthermore, other comparative studies with larger samples may be necessary to corroborate these data and evaluate the safety of carbamazepine.

## CONCLUSION

Carbamazepine failed to prevent CINV and also presented a deleterious side-effect profile among the patients studied. Based on these data, carbamazepine is not advisable as an antiemetic medication for patients undergoing chemotherapy outside of experimental settings. 
